# Reliability and validity of Healthy Fitness Measurement Scale Version1.0 (HFMS V1.0) in Chinese elderly people

**DOI:** 10.1186/s12889-021-11021-2

**Published:** 2021-05-30

**Authors:** Qian Liu, Hui Zhou, Heng Qiu, Chen Huang, Lijie Jiang, Guli Jiang, Weixuan Wu, Zhuomin Huang, Jun Xu

**Affiliations:** 1 Department of Sanitation Economy Administration, Nanfang Hospital, Southern Medical University, Guangzhou, Guangdong Province China; 2 School of Health Management, Southern Medical University, Guangzhou, Guangdong Province China; 3 Guangzhou Cadre Health Management Center, Guangzhou, Guangdong Province China; 4 Guangdong Provincial Occupational Disease Prevention and Control Hospital, Guangzhou, Guangdong Province China

**Keywords:** Healthy fitness measurement scale version 1.0 (HFMS V1.0), Chinese elder people, Reliability, Validity

## Abstract

**Purpose:**

We examined the reliability and validity of the Healthy Fitness Measurement Scale Version 1.0 (HFMS V1.0) specifically on elderly people in China.

**Methods:**

We carried out a cross-sectional study in December 2020 and enrolled 800 elderly people through stratified sampling technique, including 777 valid samples (with a mean age of 71.81 ± 8.36 years), of which 382 cases (49.2%) were women. The level of healthy fitness was measured using the HFMS V1.0. The Cronbach’s alpha coefficient, split-half reliability, test-retest reliability, convergent and discriminant validity, exploratory factor and confirmatory factor were calculated for assessing the reliability and validity of HFMS V1.0.

**Results:**

HFMS V1.0 consists of 8 dimensions and 38 items. The scale had acceptable reliability (Cronbach’s alpha = 0.920, split-half = 0.946, test-retest = 0.878). Exploratory factor analysis showed KMO value =0.927, and uncovered 10 factors with the cumulative contribution rate of 65.71% and all factor loads over 0.40. The item distribution was consistent with the initial expectation of the scale. The confirmatory factor analysis indicated good fit: CMIN/DF = 2.796, RMSEA = 0.048, IFI =0.914, TLI = 0.902, CFI = 0.913.

**Conclusion:**

HFMS V1.0 was shown to have acceptable reliability and validity indices for this sample. Collectively, HFMS V1.0 is reliable and efficient to measure the healthy fitness of elderly people. It is recommended to use it among the elderly in other Chinese cities in the future to ensure uniformity and objectivity. This scale can be carried out to evaluate of the effectiveness of public health measures in improving the healthy fitness level of the elderly and optimizing public health policies.

## Introduction

The increase in life expectancy and the decline in fertility are facilitating the aging of the world’s population [1]. By the end of 2019, China’s elderly population has exceeded 250 million, accounting for 18.1% of the total population [2]. By 2053, China’s elderly population will reach the peak of population aging. From 2000 to 2050, the ratio of China’s population aging will increase from 10 to 34%, over two times as the global growth rate [3]. The disease spectrum in China has begun to transit from infectious diseases to non-communicable diseases. The prevalence of chronic non-communicable diseases will increase by at least 40% by 2030, when approximately 80% of people aged 60 and over will die from chronic non-communicable diseases. Whether an aging population can create a “third demographic dividend” for society depends heavily on the health [4]. Biological aging is characterized by physical weight loss [5], decline in organ function [6], and psychological memory [7], emotional instability, and reduced adaptability [8]. Due to reduced adaptability in many aspects, the prevalence of chronic diseases in the elderly is 2.3–3.2 times that of the total population [9]. Therefore, it is imperative to strengthen the healthy fitness management of the elderly. The concept of “health” (“Health is not only the absence of disease and infirmity, but also a state of physical, mental and social well-being”) was introduced by WHO in 1947 [10]. Fitness refers to the individual’s ability to actively or passively adapt to changing environment, including all physical, mental and social responses [11]. Sturmberg proposed that the dynamic adaptive relationship between the individual and internal and external factors determines the state of health [12]. There is an increasing body of evidence supporting that fitness levels relate to current and future individuals’ health status [13]. A high level of fitness allows people to effectively cope with the internal and external events and to restore to a balanced state following stress reaction and adjustments; otherwise, individual with poor fitness are more vulnerable to health impacts of external forces and even many diseases [14]. Fitness is an indispensable ability for people living in modern society, also an important factor in health [15].

Physical fitness is the ability to cope with daily work without undue fatigue, and with energy to enjoy leisure and respond to emergencies [16]. Physical fitness is closely related to health-related quality of life [17]. Studies have shown that good Physical fitness can have a protective effect against certain cancers and reduce the risk of cardiovascular disease and metabolic syndrome [18]. Physical exercise is an important way to enhance healthy physical fitness [19]. By measuring the physical fitness status of individuals, it can effectively guide them to participate in physical exercise and develop healthy awareness and behavior. It is now common practice in some countries to develop and implement various physical fitness testing and evaluation standards. The physical fitness test in the United States was traced back to the 1880s [20]. The Physical Best (PB) is currently prevalent test for assessing physical fitness [21], whose selected indicators consist of cardiopulmonary function, muscle strength and endurance, flexibility and body composition. The Japanese physical fitness tests are made up of different test items based on age and grade, with grip strength, sit-ups and sit-and-reach as general items [22, 23]. China’s physical fitness test was developed late, and the test follows *National Fitness Standards* covering all people different ages (from infants, children and adolescents, adults to the elderly) [24]. However, the above-mentioned tests rely on professional assessors and are limited by the availability of space and equipment. At the same time, subjects with sudden illnesses such as colds, sports injuries, and cardiovascular diseases as “exemptions” in the testing process [25], so that individuals with weak economic and health conditions cannot know their own physical fitness level. Therefore, it is necessary to develop a convenient, effective, and reliable fitness self-assessment tool to allow individuals to understand their own physical fitness status in real time and to guide them to effectively carry out physical fitness promotion activities.

Martin Prince emphasizes the separate contributions of mental and physical illness to disability and mortality, and suggests that there is no health without mental health [26]. The epidemic of psychosocial distress and mental ill health have become major threats to people’s well-being [27]. Therefore, mental fintess is also an important aspect of healthy fitness assessment. Mental fitness is defined as the interaction between an individual and a changing environment, and it is a dynamic process of individual psychological self-regulation [28]. Mental fitness is closely related to disease progression and people’s physical health [29]. Paula Robinson [30] describes mental fitness as the capacity to use one’s resources and skills to flexibly adapt to environmental changes, and proposes that mental fitness can be measured, when the mental fitness can be understood in a similar way to physical fitness. Linda Bolier [31] pointed out that positive thinking and problem-solving capacity has a positive effect on health. Different from the quantitative assessment of physical fitness using instruments and equipment, evaluation of mental fitness at home and abroad is mostly carried out by the scale or the evaluation index system, such as Adolescence Psychological Adaptability Scale (APAS) [32] for evaluating the psychological adaptability of adolescents, Symptom Check List-90 (SCL-90) [33] for evaluating mental health status, and Self-Rating Anxiety Scale (SAS) for evaluating psychological anxiety [34].

Social fitness is defined as the ability of individuals to adjust their own body and psychological state to achieve the goals expected by the society [35], particularly encompassing the availability and compatibility of social environment [36]. This kind of fitness is affected by both internal and external factors [37]. Individuals with lower level of social adaptability are more prone to maladaptation with symptoms such as fear and cringe, and even environmental shock [38–40]. At present, there have been some researches on the measurement of individual social adaptiveness and adaptability at home and abroad, such as Vineland Social Maturity Scale (VABS) [41] and the Social Adaptation Self-evaluation Scale (SASS) [42], American Association for Mental Deficiency Adaptive Behavior Scales (AAMD ABS) [43], and Psychosomatic Symptom Scale (PSSS) [44].

Fore-mentioned studies on adaptability evaluation mostly focus on a certain aspect of fitness other than integration of physical, mental and social fitness. In 1948, World Health Organization (WHO) defined health as a state of the absence of illness or weakness, and the presence of physical, psychological, and social well-being [45], discarding the narrow concept of “health”, but encompassing psychological and social well-being. Social competence and adaptability have become essential to health. Therefore, comprehensive assessment of adaptability should not only include measures of physical fitness (health-related physical fitness), but also detect mental and social fitness [46]. On the basis of previous studies on physical fitness and health-related physical fitness, our previous study put forward with the concept of “healthy fitness” [47]: the best physical, mental and social adaptability. Further, Jun Xu et al. established a healthy fitness assessment index system of Healthy Fitness Measurement Scale Version 1.0 (HFMS V1.0) involving physical, mental and social fitness when considering China’s social culture [48] .Herein, our study aimed to determine the reliability and validity of HFMS V1.0 applied to the elderly, and provide a convenient and efficient self-reporting tool for health fitness evaluation, allowing medical institutions and public health practitioners to conduct targeted behavioral interventions and health guidance for the elderly to prevent harmful effects secondary to decreased health fitness.

## Materials & methods

### Study design

This a cross-sectional and multistage survey was conducted using a random sampling technique in December 2020. The first stage involved 4 administrative districts within Guangzhou while considering their economic level and geographical distributions. The second stage involved 1 ~ 3 streets of the selected districts. The final stage involved sampling of 1 ~ 2 neighborhood committees from the selected streets. Finally, elderly people in eight elderly care institutions and community hospitals in four administrative districts (Huangpu, Yuexiu, Liwan, Baiyun) of Guangzhou city were included in this study. These facilities were selected mainly due to their location close to our institution. For subjects who met our inclusion criteria and agreed to participate in our study, the random sampling was conducted based on gender (male: female = 1:1) and age ((60–64):(65–69):(70–74):(75–79):(80 years and older) = 1:1:1:1:1:1).

### Participants

The sample size was at least 10–15 individuals per item for the factor analysis. If the sample size was more than 20 individuals per item for the factor analysis, the results of factor analysis would be more stable and reliable [49]. The sample size was calculated as 680 with 20 individuals each item. Considering shedding, the final sample size was set to 800. All participants completed the test and 80 of them participated in the retest over an interval of 24 h to 1 week. Inclusion criteria included the following: age over 60 years old, local residents or non-local residents who have lived for more than half a year, and willingness to participate in this survey. Exclusion criteria were cognitive decline and a history of illness within this month. In the first test, 777 valid questionnaires (male, 50.8%) were returned, with effective response rate of 97.13%. Seventy-four valid questionnaires were retested and returned, with effective recovery rate of 92.50%. Informed written consent was obtained from all subjects.

### Healthy fitness assessment

A number of sociodemographic variables were set in this study, including: gender, age, educational background, marital status, household monthly income, personal monthly income, pre-retirement occupation, participation in insurance. The age range was classified into five groups: “60 to 64 years old”, “65 to 69 years old”, “70 to 74 years old”, “75 to 79 years old”, and “over 80 years old”. The educational background of the respondents was categorized as: “uneducated”, “primary school diploma”, “junior high school diploma”, “high school/technical secondary school/vocational high school diploma”, “college degree”, “bachelor degree and above”. The marital status was classified into five groups: “single”, “married”, “divorced”, “widowed”, “others”. The household monthly income of the respondents was categorized as: “RMB 3000-RMB 6000”, “RMB 6001-RMB 9000”, “RMB 9001-RMB 12000”, “Over RMB 12001”. The personal monthly income of the respondents was categorized as: “Less than RMB 2000”, “RMB 2001-RMB 4000”, “RMB 4001-RMB 6000”, “RMB 6001-RMB 8000”, “Over RMB 8001”. The pre-retirement occupation was indicated as: “Heads of state agencies, party organizations, enterprises, and institutions”, “Professional technicians (teachers, doctors, etc.)”, “Clerks and related personnel”, “Commercial and service personnel”, “Production personnel in agriculture, forestry, animal husbandry, fishery and water conservancy”, “Production, transport and equipment operators and related occupations”, “Soldier”, “Other practitioners”. The participation in insurance was denoted as: “Self-pay”, “Public medical insurance”, “Medical insurance for urban and rural residents”, “Urban employee medical insurance”, “Commercial medical insurance”.

Healthy fitness was the adaptability outcome analyzed in this study. This was measured using the Health Measurement Scale version 1.0 (HFMS V1.0), which had been previously developed by our research group. This scale conforms to operational definition of healthy fitness and has been analyzed and confirmed by the expert and field investigation [48]. HFMS V1.0 consists of three subscales: physical fitness status (PF), mental fitness status (MF), and social fitness status (SF). PF consists of 14 items that comprises three factors: organic function, motor function and physical adaptive capacity. MF consists of 11 items that comprises three factors: psychological cognition, resilience and stress response. SF consists of 9 items that comprises two factors: role adaptation and social resource and social support. Forward scoring must be adopted for the 1–5, 16–17, 28–36 with the score equal to the original score, while reverse scoring (6–1) must be adopted for the items 6–14, 18–26, and items 15, 27, 37, and 38 were the overall evaluation items and not calculated. The scale used Likert 5-point method (1 = very poor, 2 = poor, 3 = moderate, 4 = good, 5 = very good). The original score of each dimension was computed as the sum of the scores of each subordinate items, and the original score of each subscale was computed as the sum of the scores of each subordinate dimensions. The gross score of the scale was computed by the sum of the scores of the three subscales. For better analysis, comparison, and popularization, the raw scores of each dimension and each scale are converted to percentile value with formula as follows. The higher the conversion score, the higher the fitness level [48].

$$ Conversion\kern0.6em score=\frac{Original\kern0.36em score- Theoretical\kern0.34em Maximum}{Theoretical\kern0.34em Maximum- Theoretical\kern0.34em Maximum}\ast 100 $$

### Quality control

The uniformly trained investigators sent out the questionnaire to the subjects, and introduced the filling method and precautions. The subjects were required to respond independently and completed the questionnaire by themselves based on their own healthy fitness in the past month. If the participants have trouble in reading the questionnaires, the investigator may provide appropriate assistance to them without any inducing prompts. In order to ensure the quality of the questionnaires, all questionnaires were collected on the spot, and those with more than 6 missing items, inconsistent answers, regular answers, or highly repeated answers were excluded.

### Statistical analysis

Missing values are filled using multiple interpolation(m = 5) [50]. All data were processed by IBM SPSS 25.0 software and AMOS 21.0 software. Quantitative data were described as (^−^X ± S) and count data were described as percentage. Reliability denotes the ability of a scale to produce consistent results when completed under similar conditions, whereas validity denotes the extent to which a scale measures the construct it is supposed to. Reliability of the questionnaire as internal consistency was determined using split-half method and Cronbach’s alpha coefficient. Cronbach’s alpha coefficient of 0.81 to 1.00 indicates almost perfect agreement, 0.61 to 0.80 indicates agreement, 0.41 to 0.60 indicates moderate agreement [51]. Split-half method reliability was assessed by calculating the 34 odd- and even-numbered items after removing 4 overall items not involved in scoring, with its coefficient over 0.70 considered satisfactory [52]. The intraclass correlation coefficient (ICC) was calculated for evaluating test-retest reliability with values less than 0.5, between 0.5 and 0.75, between 0.75 and 0.9, and greater than 0.90 indicative of poor, moderate, good, and excellent reliability, respectively [53]. Validity was evaluated using convergent and discriminant validity, as well as factor analysis consisting of exploratory factor analysis (EFA) and confirmatory factor analysis (CFA). Examination of convergent and discriminant validity included evaluation of spearman’s correlation coefficient [54]. In general, great convergent and discriminant validity is characterized by the correlation coefficient between each dimension value and the total value higher than that between each dimension; the correlation coefficient between each dimension and the scale’s total score > 0.40 [55]. For EFA, we used the Kaiser-Meyer-Olkin (KMO) test and Bartlett’s test of sphericity to measure the adequacy of samples when determining whether KMO value is between 0.5 and 1 [56]. Principal components analysis (PCA) was used to obtain common factors. In order to determine the factor structure, the orthogonal rotation axis was performed by the varimax rotation. CFA was performed to assess the measurement model. Good model fit [57] included chi-square (CMIN/DF) < 3.00, root mean square error of approximation (RMSEA) < 0.05, incremental fit index (IFI) > 0.900, Tucker-Lewis index (TLI) > 0.900, comparative fit index (CFI) > 0.900. * *p* < 0.05 indicates significant difference.

## Results

### Description of sample

The demographics of all participants are shown in Table 1. Of the 777 participants, males accounted for a larger proportion (50.8%) with most in the 65–69 age group (26.1%). Education of the most participants was junior high school and above (76.2%), and most of the participants were married (79.9%). Their pre-retirement occupations were mainly heads of state agencies, party organizations, enterprises, and institutions (34.2%), and a majority participated in public medical insurance (57.1%).

**Table 1:** Participant’s demographic characteristics (*n* = 777)

Characteristic	Number	Percent
Gender		
Male	395	50.8
Female	382	49.2
Age (years old)		
60–64	164	21.1
65–69	203	26.1
70–74	139	17.9
75–79	108	13.9
80-	163	21.0
Education		
Uneducated	43	5.5
Primary school diploma	142	18.3
Junior high school diploma	132	17.0
High school/technical secondary school/vocational high school diploma	207	26.6
College degree	174	22.4
Bachelor degree and above	79	10.2
Marital status		
Single	14	1.8
Married	621	79.9
Divorced	35	4.5
Widowed	101	13.0
others	6	0.8
Household monthly income per person (yuan)		
< 3000	95	12.2
3000–6000	262	33.7
6001–9000	187	24.1
9001–12,000	133	17.1
> 12,000	100	12.9
Personal monthly income (yuan)		
< 2000	55	7.1
2000–4000	124	16.0
4001–6000	239	30.8
6001–8000	137	17.6
> 8000	222	28.6
Pre-retirement occupation		
Heads of state agencies, party organizations, enterprises, and institutions	266	34.2
Professional technicians (teachers, doctors, etc.)	113	14.5
Clerks and related personnel	127	16.3
Commercial and service personnel	71	9.1
Production personnel in agriculture, forestry, animal husbandry, fishery and water conservancy	59	7.6
Production, transport and equipment operators and related occupations	39	5.0
Soldier	4	0.5
Other practitioners	98	12.6
Participation in insurance		
Self pay	25	3.2
Public medical insurance	402	51.7
Medical insurance for urban and rural residents	189	24.3
Urban employee medical insurance	235	30.2
Commercial medical insurance	50	6.4

### Validity

#### Exploratory factor analysis

Table 2 showed the descriptions of each item. Since Skewness and Kurtosis values are all less than 2, all of the items were retained in the exploratory factor analysis.

**Table 2:** The descriptions of each item of the HFMS V1.0 (*N* = 777)

Subscale	Dimension	Item	Mean	SD	Skewness	Kurtosis
Physical Fitness Subscale	Organic Function	1. Shapely	3.02	0.82	− 0.26	0.08
		2. vision	2.93	0.78	−0.13	− 0.20
		3. hearing	3.14	0.82	−0.42	− 0.17
		4. Head discomfort	3.45	0.85	−0.31	0.23
		5. Palpitation	3.62	0.87	−0.18	−0.10
	Motor Function	6. Climb 3–5 floors	2.92	0.90	0.17	0.04
		7. 1000 m walk	3.23	1.10	0.03	−0.81
		8. Bend over to touch toes	2.79	1.04	0.17	−0.52
		9. Daily housework	3.30	0.88	−0.03	− 0.05
		10. Participate in strenuous activities	2.63	0.91	0.25	−0.13
	Physical Adaptive Capacity	11. Easy to catch colds and allergies	3.18	0.78	0.08	−0.13
		12. Noise, light interference	2.99	0.86	0.16	−0.17
		13. Cold resilience	3.26	0.79	−0.17	− 0.20
		14. Relieve discomfort	3.43	0.87	−0.23	−0.48
Mental Fitness Subscale	Psychological Cognition	16. Focus	3.25	0.78	−0.15	−0.46
		17. memory	2.81	0.78	0.16	−0.48
	Resilience	18. Strive to achieve the goal	3.25	0.78	0.00	0.14
		19. Drop goal	3.24	0.79	0.18	0.45
		20. There is hope in the future	3.39	0.82	0.12	−0.29
		21. Discouraged by failure	3.44	0.77	0.21	0.05
	Stress Response	22. loneliness	3.71	0.90	−0.10	− 0.66
		23. feeling scared	3.87	0.85	−0.28	− 0.53
		24. upset	3.56	0.75	0.15	−0.39
		25. Restless	3.75	0.81	−0.07	− 0.60
		26. nervous	3.60	0.75	0.07	−0.38
Social Fitness Subscale	Role Adaptation	28. Family relations	3.55	0.82	−0.56	0.49
		29. Unpleasant handling	3.55	0.80	−0.39	0.11
		30. Adapt to role changes	3.49	0.91	−0.31	− 0.40
		31. Self-role evaluation	3.55	0.80	−0.46	0.25
	Social Resource and Social Support	32. Connect with relatives and friends	3.41	0.81	−0.02	− 0.37
		33. Support from relatives and friends	3.29	0.82	−0.23	0.01
		34. Proactively seek help	2.92	0.80	0.08	0.06
		35. Share with others	3.18	0.84	0.18	−0.21
		36. Close friends	3.33	0.85	0.11	−0.13
Overall evaluation item		15. Overall evaluation of physical fitness	3.23	0.68	−0.07	0.00
		27. Overall evaluation of mental fitness	3.28	0.72	−0.10	0.08
		37. Overall evaluation of social fitness	3.45	0.71	−0.15	0.08
		38. Overall assessment of healthy fitness	3.42	0.70	−0.17	0.34

**Fig. 1:**
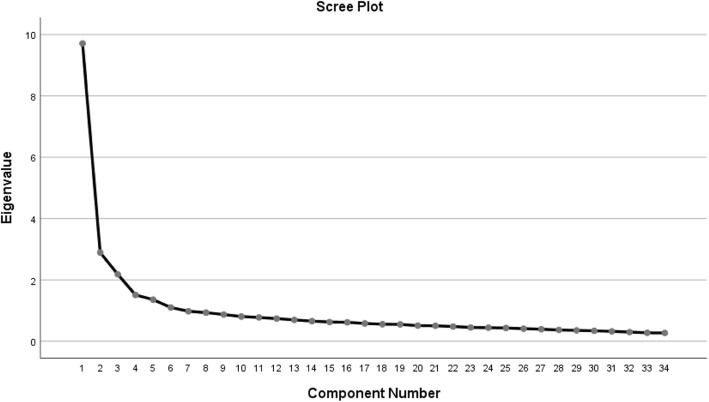
The Scree plot of HFMS V1.0

A KMO test was used in research to determine whether the sampling adequacy of data are to be used for factor analysis. As a consequence, the data of high KMO value (0.927) demonstrated that a factor analysis may be useful; meanwhile the approximate chi-square distribution of Bartlett test was 10,646.015, the degree of freedom was 561, (*p* < 0.001), refuting the hypothesis that the correlation matrix is not an identity matrix. This indicates that 34 items have common factors and therefore are suitable for factor analysis [58]. In the PCA, Combined scree plot (Fig. 1), factor loading matrix and previous theoretical inferences, 10 factors were extracted and the cumulative contribution rate reached 65.71%. Table 3 showed the item distribution of the 10 factors was roughly in line with the theory of scale compilation with the factor loads higher than 0.4 after the orthogonal rotation axis is performed by the varimax rotation (factor 1: Motor function; factor 2: Role adaptation; factor 3: Stress response; factor 4: Resilience; factor 5 and factor 7: Organ function; factor 6 and factor8: Social resource and social support; factor 9: Physical adaptive capacity; factor10: Psychological cognition).

**Table 3:** Factor loading matrix and contribution rate of each factor

	Factor1	Factor2	Factor3	Factor4	Factor5	Factor6	Factor7	Factor8	Factor9	Factor10
HF1	0.408				0.447					
HF2					0.797					
HF3					0.698					
HF4							0.709			
HF5							0.607			
HF6	0.750									
HF7	0.751									
HF8	0.664									
HF9	0.654									
HF10	0.810									
HF11	0.439								0.523	
HF12									0.581	
HF13									0.504	
HF14									0.422	
HF16										0.701
HF17										0.655
HF18				0.686						
HF19				0.762						
HF20				0.671						
HF21			0.428	0.622						
HF22			0.492	0.424						
HF23			0.732							
HF24			0.782							
HF25			0.754							
HF26			0.715							
HF28		0.529								
HF29		0.706								
HF30		0.788								
HF31		0.757								
HF32		0.500								
HF33		0.442						0.652		
HF34								0.838		
HF35						0.825				
HF36						0.690				
Contribution rate (%)	28.558	8.508	6.416	4.441	3.993	3.235	2.878	2.743	2.564	2.374
Cronbach’s alpha coefficient	0.843	0.804	0.839	0.790	0.623	0.764	0.573	0.532	0.719	0.550

Factor loading>0.4

### Confirmatory factor analysis

**Fig. 2:**
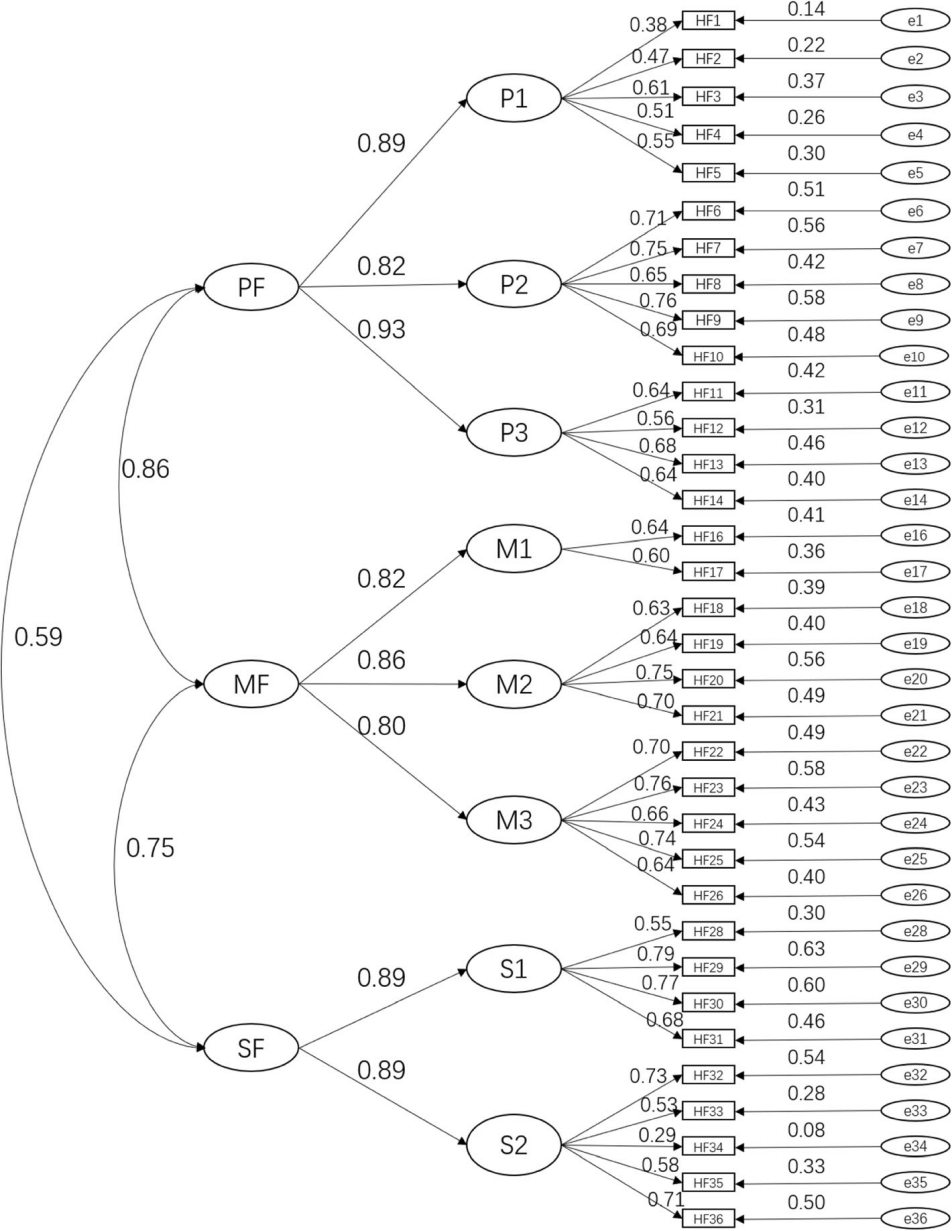
The revised overall model of HFMS V1.0. Notes: The scale entries (HF1-HF36) were observed variables. P1, P2, P3, M1, M2, M3, S1, S2, PF, MF, SF were potential variables, where P1, P2, P3, M1, M2, M3, S1, S2 were endogenous variables and PF, MF, SF were exogenous variables. PF=Physical Fitness subscale, MF = Mental Fitness subscale, SF=Social Fitness subscale. P1 = Organ function, P2 = Motor function, P3 = Physical adaptive capacity, M1 = Psychological cognition, M2 = Resilience, M3 = Stress response, S1 = Role adaptation, S2 = Social resource and social support

Combined with the secondary structure of the HFMS V1.0 scale, a second order CFA structure was modeled, as shown in Fig. 2. The correlation coefficients among the three subscales of PF, MF, and SF were 0.59, 0.86, 0.75, and the standardized path coefficients between the dimensions and the subscales ranged from 0.78 to 0.95. The path coefficients of most items over 0.50 indicated that HFMS V1.0 has a large effect with great path association. The initial model was not well fitted (CMIN/DF = 3.647, RMSEA = 0.058, IFI =0.867, TLI = 0.855, CFI = 0.867.), so covariation relationship between the error variables was established in turn by combining the Modification Indices and Estimated parameter change for covariance. After the correction, the model showed good fit: CMIN/DF = 2.796, RMSEA = 0.048, IFI =0.914, TLI = 0.902, CFI = 0.913.

The calculated correlation coefficients between dimensions and subscales score had a range of 0.597–0.886, showing that the dimensions and subscales scores had good convergent validity. Additionally, the correlation coefficients among the dimensions were lower than the correlation coefficients between the dimensions and the corresponding subscale score, which indicated that the scale had good discriminant validity (Table 4).

**Table 4:** Correlation coefficient matrix between dimensions and subscales

	Organ function	Motor function	Physical adaptive capacity	Psychological cognition	Resilience	Stress response	Role adaptation	Social resource and social support
Organ function	1							
Motor function	.485**	1						
Physical adaptive capacity	.480**	.611**	1					
Psychological cognition	.388**	.404**	.374**	1				
Resilience	.432**	.438**	.513**	.442**	1			
Stress response	.490**	.340**	.480**	.343**	.572**	1		
Role adaptation	.410**	.372**	.358**	.358**	.444**	.501**	1	
Social resource and social support	.248**	.226**	.170**	.296**	.338**	.278**	.527**	1
Physical fitness subscale	.759**	.882**	.809**	.467**	.551**	.511**	.442**	.257**
Mental fitness subscale	.550**	.471**	.573**	.597**	.840**	.886**	.552**	.366**
Social fitness subscale	.353**	.315**	.278**	.357**	.438**	.431**	.846**	.886**

**significant, *p*<0.001. The bold correlation coefficient is the correlation coefficient between each dimension and the corresponding subscale

#### Reliability

The Cronbach’s alpha of HFMS V1.0 scale was 0.920, and the Guttman coefficients of the HFMS V1.0 total scale was 0.946. Three subscales reliability results, means and standard deviations are provided in Table 5. The highest and lowest scores accounted for very low proportions in the HFMS V1.0 total scale and the three subscales of PF, MF, and SF, without ceiling and floor effect.

**Table 5:** Internal consistency reliability results, means and standard deviation, floor and ceiling effects of the HFMS V1.0 (*N* = 777)

Scale	Cronbach’s alpha	Guttman coefficient	Mean	SD	Floor (%)	Ceiling (%)
HFMS V1.0	0.920	0.946	57.37	11.00	15.44(0.13%)	91.91(0.13%)
PF	0.869	0.884	53.37	13.37	12.50(0.13%)	92.86(0.13%)
MF	0.865	0.893	61.07	13.04	13.64(0.13%)	100.00(0.13%)
SF	0.853	0.905	59.09	13.48	19.44(0.26%)	94.44(0.37%)

*PF* physical fitness subscale, *MF* mental fitness subscale, *SF* social fitness subscale. *SD* standard deviation

All HFMS items exhibited satisfactory correlation with the corresponding subscale scores (Spearman’s r > 0.30) and ranged from 0.421 to 0.724. The Cronbach’s α values were above the threshold of 0.70 and ranged from 0.803 to 0.869(Table 6).

**Table 6:** Item-Total Correlation and Cronbach’s Alpha of three subscale (*N* = 777)

Item	Item-Total Correlation	Cronbach’s Alpha if Item Deleted
Physical Fitness subscale		
HF1	.425**	0.869
HF2	.476**	0.866
HF3	.565**	0.861
HF4	.478**	0.866
HF5	.530**	0.865
HF6	.647**	0.854
HF7	.705**	0.853
HF8	.646**	0.857
HF9	.724**	0.851
HF10	.663**	0.856
HF11	.629**	0.858
HF12	.537**	0.862
HF13	.612**	0.859
HF14	.613**	0.859
Mental Fitness subscale		
HF16	.516**	0.865
HF17	.486**	0.865
HF18	.580**	0.856
HF19	.618**	0.854
HF20	.689**	0.850
HF21	.703**	0.848
HF22	.720**	0.848
HF23	.703**	0.850
HF24	.653**	0.853
HF25	.724**	0.847
HF26	.628**	0.855
Social Fitness subscale		
HF28	.635**	0.819
HF29	.687**	0.807
HF30	.687**	0.808
HF31	.682**	0.808
HF32	.705**	0.806
HF33	.641**	0.816
HF34	.421**	0.840
HF35	.655**	0.811
HF36	.720**	0.803

Table 7 shows the test-retest reliability statistics in older adults from Guangzhou for the HFMS V1.0 and three subscale: PF, MF, and SF. The ICC values ranged from 0.752 (SF) to 0.837 (MF), and the ICC of HFMS V1.0 was 0.878.

**Table 7:** Test–retest (24 h to 1 week apart) reliability of the HFMS V1.0 among older adults in Guangzhou (*n* = 75)

Scale	Test mean (SD)	Re-test mean (SD)	ICC	95% CI	α
HFMS V1.0	60.79(9.10)	59.59(8.73)	0.878	0.807–0.923	0.881
PF	57.48(10.14)	56.20(10.22)	0.797	0.679–0.871	0.798
MF	63.88(10.83)	61.22(13.11)	0.837	0.735–0.899	0.847
SF	62.16(12.51)	62.89(8.73)	0.752	0.608–0.844	0.751

*PF* physical fitness subscale, *MF* mental fitness subscale, *SF* social fitness subscale, *SD* standard deviation, *ICC* intraclass correlation coefficient, *CI* confidence interval, *α* Cronbach’s alpha

## Discussion

The increase in life expectancy and the decline in fertility are facilitating the aging of the world’s population [1]. In order to promote healthy aging, the WHO has released the *World report on ageing and health*, emphasizing that fitness is related to health, which hinges on the intrinsic capacity of the individual and environmental characteristics [59]. However, the current assessment of fitness is mostly limited to a certain dimension of physiology, psychology and society, and there is a lack of comprehensive healthy fitness measurement approaches. In this study, we aimed to assess the reliability and validity of the HFMS V1.0 for measuring the healthy fitness of the elderly.

Our results demonstrated that HFMS V1.0 scale exhibits acceptable internal consistency (Cronbach’s alpha coefficient = 0.920, split-half coefficient = 0.946 > 0.70), which is consistent with data of previous findings (Cronbach’s alpha coefficient = 0.920, split-half coefficient = 0.763) [48]. This indicates that all items in the HFMS V1.0 scale have good correlation with similar feature. The test-retest reliability of HFMS V1.0 scale was evaluated through examination of ICC value, as the result of ICC = 0.878 confirms the scale stability over time. In the study, the highest and lowest scores accounted for very low proportions in the HFMS V1.0 total scale and the three subscales of PF, MF, and SF. No ceiling effect or floor effect was observed in the HFMS V1.0, indicating that these aggregate scores sensitively reflect the changes in the healthy fitness of the Chinese elderly.

The test on convergent validity of the HFMS V1.0 scale indicates the strong correlation (r = 0.597–0.886) between each dimension and subscales. In the test on discriminant validity, the correlation coefficient between each dimension value and the total value was higher than that between each dimension, which indicates great convergent and discriminant validity of the HFMS V1.0 scale.

Besides, the results from EFA and CFA further depict the factorial validity of the HFMS V1.0 scale. In the EFA, the extracted 10 factors are responsible 65.71% of the variability. Among the 10 factors, factor 1 (motor function) accounts for 28.56% of the variability, suggesting that individual’s motor function should deserve more attention. Different from our conclusion, a previous study by Lijie Jiang [48] points out stress response as the main impact factor responsible for 9.485% of the variability. Such difference may be due to the compositions of the subjects; the subjects of our study were retired elderly while Li’s research focused on civil servants, most of whom were under 30 years old (47.5%). Physical fitness is related to age. The decline in function activity of skeletal muscle [60] affects the balance and walking ability of the elderly [61]. It is noted that physical fitness reaches a peak at the age of 20 [62]. Civil servants are more available to mental disorders. According to relevant data, 33.8% of civil servants suffers from high work pressure [63], while some 47% of compensatory mental disorders are triggered by work pressure [64]. Individuals under long-term stress are prone to psychological discomfort and negative emotional reactions [65]. The stress response is significantly related to psychological health [66].

In the CFA, we set up a second-order factor model to examine the scale fitness based on theoretical structure of the HFMS V1.0 scale. The standardized path coefficients between the dimensions and the subscales ranged from 0.78 to 0.95 indicating HFMS V1.0 has great path association. The initial model failed to indicate acceptable fitness. But after adjustment of fixed parameters and establishment of covariation relationship between the error terms based on the MI value and the estimated parameter change, the overall model of the scale indicated good fitness (CMIN/DF = 2.796, RMSEA = 0.048, IFI =0.914, TLI = 0.902, CFI = 0.913).

The main advantage of HFMS V1.0 scale is comprehensive evaluation of healthy fitness with systematic structure as the scale involves examinations of physical, mental fitness, and social fitness. Our study first confirms the reliability and validity of HFMS V1.0 in the Chinese elderly population through EFA and CFA, when describing the operational definition of healthy fitness.

### Limitations

Firstly, all the data in this study were collected from questionnaires filled out by the subjects of the elderly with diminished cognitive abilities, so there might exist certain potential reporting biases. Secondly, the self-report method was adopted through which the participants made an evaluation of their health fitness in the past month, but there may be a recall bias. Besides, we used a multi-stage stratified sampling method and sampling errors are still inevitable. Though the present study provides evidence for effective application of HFMS V1.0, the survey sampling was limited to four regions of the city of Guangzhou. Large-scale investigations and empirical studies should be further conducted in China in the future.

### Generalisability

As far as we know, this study first uses HFMS V1.0 to assess the health fitness level of the elderly, but the participants in all stages of this study were selected from Guangzhou city. Therefore, what extent the study sample reflects the health condition of entire Chinese elderly population remains unknown. The HFMS V1.0 should be tested among the elderly from different regions of China, thereby contributing to nationwide application of the scale. Additionally, considering cultural differences between different countries, the use of this scale in other countries requires a further cross-cultural revision and verification.

## Conclusion

This study confirms that the HFMS V1.0 scale has acceptable reliability and validity in the assessment of the healthy fitness of the elderly in Guangzhou, and it can be used as an effective and reliable quantitative measurement of the healthy fitness level of the elderly in other regions of China. These evidences might lay a good foundation for further research on the healthy fitness norms of the elderly and their related factors.

## Data Availability

Data are available upon reasonable request. Readers can contact Xu Jun (drugstat@163.com) to submit raw data access requirements.
